# Matchmaker, matchmaker, find me a match

**DOI:** 10.1120/jacmp.v16i1.5425

**Published:** 2015-01-08

This issues editorial is an invited commentary authored by John Antolak. It discusses the new CAMPEP‐accredited residency match for medical physicists. This has been a difficult effort and all involved in this effort deserve our thanks and gratitude.

Michael D. Mills, PhD

Editor‐in‐Chief

For those of you who don't identify with the title of this editorial, it is from the lyrics of a song in the 1964 Broadway musical “Fiddler on the Roof”, which was adapted into a successful motion picture in 1971.^(1)^ You may wonder what a story about Russian peasants in the late 19^th^ century has to do with medical physics today. When I was asked to write an editorial about the 2015 MedPhys Match (MPM),^(2)^ that line came to mind almost immediately. One of the main threads in the story is about finding suitable spouses for the main character's three daughters. At that time, it was common practice in some cultures to employ the assistance of a matchmaker to find a suitable spousal match. Taking into account preferences of all eligible parties, the matchmaker would propose spousal matches. Although one can argue that such an important decision should not be left to an outside party, current divorce rates and the success of online matchmaking services could be viewed as evidence to the contrary. Many articles about algorithms to solve this type of problem refer to it as the stable marriage problem.^(3)^

In my years as a medical physicist, I've witnessed many changes in the medical physics education and training landscape. The need for medical physicists has created a very rewarding (in many ways) profession, and as a result, competition to get into training programs and positions is intense. The American Board of Radiology (ABR) now requires medical physicist students to have CAMPEP‐accredited residency training to complete their board certification process. With the transition to this new requirement, there was a noticeable “food” of candidates getting into the board certification process before the new requirements took hold. I'm not going to debate the merits of the current landscape, but wanted to note that it is now here, and we have to deal with it.

Sapareto and et al.^(4)^ debated the merits of using a matching program for medical physics residency recruitment earlier in 2014 (before the MPM came into being). References in that article provide a history of how matching programs came into existence, and it is fairly obvious that there are several parallels between medical residency training recruitment (many years ago) and the current situation with medical physics residency training. As pointed out in the article, there were a variety of problems with the state of medical physics residency recruiting prior the MPM being put into place. There was an attempt at having a gentleman's agreement* between programs to provide a fair playing field for recruiting. I could give many anecdotal examples showing “bad behavior”, but as pointed out in the article, there wasn't anyone who was willing to provide any penalty for not following the agreement. The article suggests that an organization like CAMPEP could discipline programs that don't follow the agreement, but this is not within CAMPEP's mission.^(5)^

Through the efforts of too many people to name, we were able to implement the MPM to create a better recruiting environment for the 2015 recruiting season, and participation in the program is much better than we had hoped. As of late October 2014, we have more than 70 programs participating in the MPM, and more than 140 applicants have registered. The program looks like it will be a success in its first year. In some ways, program directors went out on a limb when signing up this year and I would like to thank all of them for their faith in the system.

I would like to use the rest of this editorial to deal with a few nagging questions that some might have regarding how the MPM works and whether it is really better than what we had before. First and foremost, I think that program directors and applicants worry about not being matched and that the previous system might be better in some cases. In prior years, programs would interview a few top candidates and then strike early (making an offer) to lock in the best applicant. More often than not, the applicant would accept — not because they desperately wanted to be part of that residency program, but because they were happy to get a reasonable offer. When programs race each other to find applicants, then applicants will also race each other to apply. It is pretty easy to make the argument that programs are not getting the best applicants, but a good *fast* applicant. The applicants who look the best on paper are going to get the first interviews, and hence the offers. I would argue that programs are not necessarily getting the best applicants in this case. From an applicant's perspective, they have little choice. Maybe there would have been a better offer down the road. Going back to my matchmaking analogy, a first love might be the perfect match, but many people have to play the field a little before they find their perfect match (or return to their first love).

Another common question that comes up when starting up a matching program is what happens to programs and/or applicants if they violate the match agreement. Whenever I hear that question, I always wonder if the person asking the question is thinking about violating the match agreement, but that's just the pessimist voice in my head speaking. The optimistic voice in my head says that the person asking the question is looking for assurance that their decision to follow the match agreement is a good one. National Matching Services (NMS), which is running the 2015 MPM, has indicated that match violations are rare in their experience. If NMS is made aware of any potential match violations, they will pass that information along to the MedPhys Match steering committee, which is a combined committee from SDAMPP and AAPM that is providing guidance to NMS regarding how the MPM should be run and what the terms of the match agreement are. It will be up to this steering committee to investigate any potential match violations and determine what should be done. Potential penalties might include being banned from using MPM for some period of time, reporting the violation to AAPM and/or SDAMPP for consideration or whatever else the committee might think is appropriate. While there are no specific penalties spelled out in the match agreement, the past experience of NMS is that the possibility of violations being dealt with by the sponsoring professional society is usually enough to ensure compliance. In other words, we need to trust that people want to do the right thing.

What about residency programs that traditionally take their own graduate students? This was vigorously discussed when setting up the 2015 MPM, and we came to the conclusion that it was in the best interest of the graduate students and residency programs for those residency programs to participate in the MPM. The graduate program uses the promise of a residency position (assuming satisfactory academic performance) as an incentive for graduate student recruitment, and this does not change. The residency program ranks their own graduate students at the top of their list. The graduate students are free to interview elsewhere if they desire, and they may find another residency program that they would prefer. The promise of being ranked highly by their home institution still guarantees a residency position, but because they have the opportunity to interview elsewhere, they might get an even more desirable position. The residency program can interview external candidates at the same time, and if they lose an internal candidate to another residency program, they will have the possibility of immediately filling the position with one of the external applicants.

When we first proposed the idea of doing MPM this year, diagnostic physics programs expressed considerable reticence about participating. A misconception in the argument by Sapareto et al.^(4)^ is that the diagnostic physics residency market is not large enough to participate in a matching program, but a matching program benefits both applicants and diagnostic physics residency programs. The diagnostic and therapy recruitment markets are intimately linked. If you look at applications to diagnostic physics residency programs,^†^ the vast majority of their applicants are also applying to at least a few therapy physics residency programs. I've heard diagnostic programs argue that they won't consider anyone who is also applying for therapy programs, but how can they really tell for sure? There are many applicants who would be well suited for either type of residency, and it would be a disservice to the applicants to deny them the opportunity to apply for both. Applicant and program preferences are taken into consideration by the matching algorithm, so it is very likely that diagnostic programs will be matched to applicants who have a strong preference in that area. Applicants who prefer diagnostic physics, but are not strong enough to compete for the few spots available in those programs, have a chance of landing in a possibly less desirable, but nonetheless acceptable, therapy physics residency program. Given how much modern radiation therapy relies on imaging technology, a strong argument could be made that getting strong imaging physics graduate students into therapy residencies would be a good thing.

Another argument that is often given against a matching program is that graduate students finish at all times of the year. As many graduate educators will admit, the time that a graduate student spends in the program is often dictated by the demands of the supervisor, who does not want to lose a productive member of the team. This is where graduate program directors need to step in, to end what might be considered taking advantage of the graduate student and of not acting in their best interests.^‡^ Many residency programs have a fixed schedule, which is a fact that is not likely to change. Students have caught on, and many are adjusting how they work on their research projects to get to a reasonable endpoint to coincide with the start dates of residency programs, but graduate programs need to cooperate.

The last point I would like to address is the notion that it is mandatory for all medical physics residency programs to participate in the MedPhys Match. The way we currently have it structured (subject to change), participation is voluntary and is primarily aimed at those programs that recruit for the *traditional* summer start date (approximately July 1). We have opened it up to programs with other start dates (e.g., September 1), and as long as the program is willing to live within the rules of the match agreement, it is free to participate. The rules have been developed with the assistance of NMS, based on its many years experience running such programs in other professions. The rules may change slightly from year to year under the direction of the AAPM/SDAMPP steering committee; anyone with constructive feedback is invited to contact the author privately to discuss.

In conclusion, I would again like to thank all of the hard‐working individuals who have supported the efforts to get the 2015 MedPhys Match in place. I am convinced that we are now in a better place than we were just a few years ago, and we are definitely making things better for both applicants and programs.

John A. Antolak, PhD^∫^

JACMP Associate Editor
